# Independent associations of education, intelligence, and cognition with gastrointestinal diseases and the mediating effects of risk factors: a Mendelian randomization study

**DOI:** 10.3389/fmed.2024.1342358

**Published:** 2024-02-12

**Authors:** Mingyu Gu, Minghai Wen, Di Wu, Tianyu Xie, Xinxin Wang

**Affiliations:** Department of General Surgery, The First Medical Center of Chinese PLA General Hospital, Beijing, China

**Keywords:** education, gastrointestinal diseases, Mendelian randomization, smoke, obesity

## Abstract

**Background:**

Education, intelligence and cognition affect occupational performance and socioeconomic status and may influence virous diseases development. However, the impact of these factors on gastrointestinal diseases and their mediating risk factors remains unclear.

**Methods:**

We utilized genome-wide association studies from European ancestry populations to perform two-sample Mendelian randomization analyses, aiming to estimate genetic instruments associated with education, intelligence, or cognition in relation to 24 gastrointestinal diseases Subsequently, we evaluated 14 potential mediators of this association and calculated the corresponding mediated proportions through two-step Mendelian randomization analyses.

**Result:**

As the dominant factor in gastrointestinal diseases, education had a statistically significant association with 2 gastrointestinal diseases (acute pancreatitis, gastroesophageal reflux) and a suggestive association with 6 diseases (cirrhosis, alcoholic liver disease, cholecystitis, cholelithiasis, chronic gastritis and gastric ulcer). Of the 14 mediators, smoking and adiposity traits played a major role in mediating the effects.

**Conclusion:**

The study demonstrated the causal, independent impact of education on specific gastrointestinal diseases. Smoking and adiposity traits emerged as primary mediators, illuminating potential avenues for targeted interventions for prevention of them.

## Introduction

Gastrointestinal diseases are diverse, prevalent and result in significant healthcare expenditures. Approximately 135.9 billion dollars is spent annually on healthcare for gastrointestinal diseases in the United States (1). Considering the substantial impact of gastrointestinal diseases, it is essential to investigate both their genetic and environmental determinants. Exploring these factors is imperative for the development of effective interventions and the formulation of appropriate social policies aimed at treating and preventing these diseases. This comprehensive approach holds the potential to significantly reduce the burden of gastrointestinal diseases on individuals and society as a whole. There is a strong relationship between intelligence, cognition, and education (2, 3). Higher levels of education not only provide access to knowledge and broaden horizons, but also influence the development of intelligence and cognition. These three factors are not only closely related to an individual’s occupational performance and socio-economic status, but may also influence the development and progression of a wide range of diseases in an individual (4, 5).

Numerous previous observational studies have identified educational attainment as a potential risk factor for various gastrointestinal diseases, including chronic liver diseases (6), chronic pancreatitis and pancreatic cancer (7). Conversely, some studies suggest that education may reduce the risk of certain gastrointestinal diseases such as gastric cancer (8), esophageal cancer (9). Furthermore, some gastrointestinal diseases for which the relationship with education is unclear and, it has yet to be confirmed whether these associations are indeed causal. For instance, while most studies suggest a lower incidence of cirrhosis and reduced mortality in populations with higher levels of education (10, 11), some studies have reached a contrary conclusion (6). The uncertainty arises from the reliance on evidence primarily derived from observational studies, which could be susceptible to biases stemming from reverse causation and confounding factors. Therefore, the independent causal impact of education, intelligence or cognition on gastrointestinal diseases remains unclear. Further exploration of this topic could enhance our understanding of the causes of gastrointestinal diseases and facilitate the development of strategies for disease prevention and intervention to curb disease epidemics.

Mendelian randomization is a powerful method to minimize the impact of reverse causality and confounding on causal estimates derived from observational data. It achieves this by leveraging genetic variants, which are fixed at conception and naturally randomized among individuals to serve as proxies for exposures (12). An extension of this method, known as Multivariable Mendelian randomization (MVMR), enables an equivalent analysis of mediation within a two-step Mendelian randomization framework (13).

Here, we performed an MR study to investigate the associations between education, intelligence or cognition and 24 gastrointestinal diseases. Furthermore, we extended our investigation by performing MVMR analyses to assess the potential mediating effects of relevant risk factors.

## Materials and methods

### Study design

**Figure 1:**
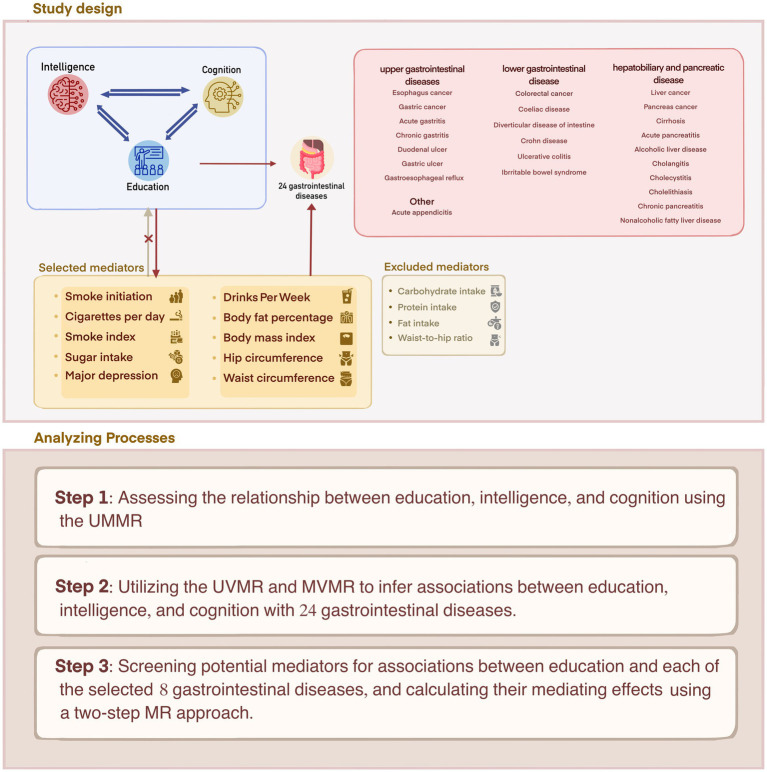
Study design overview.

Our study was conducted in three steps (Figure 1). Firstly, we evaluated the relationship between education, intelligence and cognition using the univariable Mendelian randomization (UVMR). Secondly, we used UVMR and MVMR to determine associations between education, intelligence, and cognition with 24 gastrointestinal diseases and found that only education was independently causally associated with 8 out of 24 gastrointestinal diseases. Finally, we screened candidate mediators for associations between education and each of the selected 8 gastrointestinal diseases and calculated their mediating effects using two-step MR. This study followed the guidelines for reporting observational epidemiological studies with an enhanced focus on the use of Mendelian randomization (14).

### Data sources of exposures, mediators, and outcomes

In this MR study, we obtained exposure, mediator, and outcome data from summary-level information primarily derived from genome-wide association studies (GWAS) conducted in individuals of European ancestry (Table 1). Genetic associations were estimated using data from the FinnGen study (15) and several large consortia.

**Table 1:** Sources of data in the MR study.

Phenotype	No of participants	Ancestry	Consortium/ cohort	Year of publication	Web source/Pubmed ID
**Exposure**					
Education	1,131,881	European	SSGAC	2018	30,038,396
Intelligence	269,867	European	Meta	2018	29,942,086
Cognition	257,841	European	COGENT	2018	30,038,396
**Outcome**					
Gatrointestinal diseases	—	European	FinnGen	2023	https://www.finngen.fi/fi
Irritable bowel syndrome	56,637	European	GEAR	2019	http://cg.bsc.es/gera_summary_stats/
Crohn’s disease	20,883	European	IIBDGC	2019	26,192,919
Ulcerative colitis	27,432	European	IIBDGC	2019	26,192,919
Selected mediator^*^					
**Smoke**					
Smoke initiation	632,802	European	GSCAN	2019	30,643,251
Cigarettes per day	632,802	European	GSCAN	2019	30,643,251
Smoke index	462,690	European	UKbiobank		31,689,377
**Alcohol consumption**					
DrinksPerWeek	941,280	European	GSCAN	2019	30,643,251
**Adiposity**					
Body fat percentage	454,633	European	MRC-IEU	2018	https://gwas.mrcieu.ac.uk/datasets/ukb-b-8909/
Body mass index	461,460	European	MRC-IEU	2018	https://gwas.mrcieu.ac.uk/datasets/ukb-b-19953/
Hip circumference	462,117	European	MRC-IEU	2018	https://gwas.mrcieu.ac.uk/datasets/ukb-b-15590/
Waist circumference	462,166	European	MRC-IEU	2018	https://gwas.mrcieu.ac.uk/datasets/ukb-b-9405/
**Mental disorder**					
Major depression	500,199	European	PGC	2019	30,718,901
**Excluded mediators**					
Carbohydrate intake	268,922	European	SSGAC	2020	32,393,786
Protein intake	268,922	European	SSGAC	2020	32,393,786
Fat intake	268,922	European	SSGAC	2020	32,393,786
Sugar intake	235,391	European	SSGAC	2020	32,393,786
Waist-to-hip ratio	212,244	European	GIANT	2015	https://gwas.mrcieu.ac.uk/datasets/ieu-a-73/

All GWASs have undergone ethical approval from the relevant institutional review boards, obtained informed consent from participants, and adhered to rigorous quality control protocols. Ethics approval was not necessary for this study, as it used summary-level data.

#### Exposures

We selected genetic instruments for education from a GWAS involving 1,131,881 individuals of European ancestry, carried out by the Social Science Genetic Association Consortium. Summary data were made available for 766,345 of these participants after excluding those from 23andMe due to data limitations, as data can only be reported for up to 10,000 SNPs (16). Genetic instruments for intelligence were selected from a meta-analysis of a GWAS focusing on neurocognitive tests, primarily assessing intelligence in 269,867 individuals of European descent. The analysis revealed no evidence of heterogeneity in genetic associations across cohorts (3). Genetic instruments for cognition were derived from a meta-analysis of a GWAS involving a broadband index (g) or verbal-numerical reasoning scores, encompassing 257,841 individuals from the Cognitive Genomics Consortium and UK Biobank. The analysis demonstrated low and statistically insignificant values for meta-analytic tests of heterogeneity across the studied populations (16, 17). After conducting a linkage disequilibrium analysis with a linkage disequilibrium link (r2 < 0.001; distance threshold, 10,000 kb), we identified 317, 168, and 147 independent genome-wide significant SNPs (*p* < 5 × 10^−8^) as the primary genetic instruments for education, intelligence, and cognition, respectively.

#### Mediators

Based on literature reviews, we selected 14 candidate mediators of risk factors which may lie on the pathways from education to various gastrointestinal diseases, including smoking (smoke initiation, cigarettes per day, smoke index) (18–20), dietary intake (21–24) (carbohydrate intake, protein intake, fat intake, sugar intake), Alcohol consumption (18, 25–27) (drinks per week) adiposity traits (28–31) [body fat percentage, waist-to-hip ratio (WHR), body mass index [BMI], hip circumference, waist circumference] and major depression (32–35).

Subsequently, we conducted a screening for potential mediators of the relationship between education and gastrointestinal diseases based on the following criteria: (1) Education should have a causal association with the mediator, but not the other way around. (2) The mediator should have a causal association with the outcome of gastrointestinal diseases. (3) The mediator should have a direct causal effect on the outcome of gastrointestinal diseases, independent of education. (4) Based on existing scientific evidence, the association of education with the mediator and the association of the mediator with the outcome should be in opposite directions (12).

#### Outcomes

Genetic associations with 24 gastrointestinal diseases were obtained from the FinnGen study (15), and two large consortia, including the International Inflammatory Bowel Disease Genetics Consortium (IIBDGC) (36) and Genetic Epidemiology Research on Aging (GERA) (37). The FinnGen study is a large-scale project that entails the collection and genetic analysis of data from a vast cohort, comprising over 500,000 participants drawn from Finnish biobanks. This dataset is further enriched with digital health records sourced from the Care Register for Health Care, and supplemented with valuable insights from cancer records, cause of death records, and medication reimbursement registries. The gastrointestinal endpoints were defined by ICD-8, ICD-9, and ICD-10 codes (Supplementary Table S1). We also obtained summary-level data from the IIBDGC (36) for Crohn’s disease (5,956 cases and 14,927 controls) and ulcerative colitis (6,968 cases and 20,464 controls), as well as from the GERA for irritable bowel syndrome (3,117 cases and 53,520 controls) (37).

### Statistical analysis

#### UVMR and MVMR analyses

We performed 2-sample UVMR to estimate the casual effect of each exposure (education, intelligence, and cognition) and each mediator on 24 gastrointestinal diseases, respectively. Additionally, we performed MVMR to estimate the direct effect of education, income or occupation on the outcome with mutual adjustment to determine which exposure had a causal effect independent of the other two exposures. We used the random-effect IVW method as the main analysis in UVMR and the MV-IVW method as the main analysis in MVMR. The IVW method generates the most precise, unbiased, and efficient causal estimates, assuming that the instrumental variables meet the MR assumptions (38). Estimates for each outcome from various sources were aggregated through fixed-effects or meta-analysis. False discovery rate correction was applied (value of *p*; statistical significance level < 0.05). The association with a nominal *p* < 0.05 but FDR adjusted *p* > 0.05 was regarded suggestive and the association with a FDR adjusted *p* < 0.05 was deemed significant (39).

#### Mediation MR analyses

We conducted a two-step MR analysis to investigate the potential mediating effect of an intermediate factor between education and the 24 gastrointestinal diseases (13). The first step involved estimating the causal effect (β1) of genetically determined education on the mediator using the UVMR approach. Subsequently, a Steiger analysis was performed between the mediator and education to assess the presence of bidirectionality, which could impact the validity of the mediation model. The second step was to estimate the causal effect of each mediator on each disease using MVMR with adjustment for education (β2). This analysis was based on the premise that the mediator had a causal association with the diseases, as established in the UVMR. The mediation proportion for each mediator in the relationships between education and each disease was computed as the product of β1 and β2, divided by the total effect of education on the outcome. The 95% confidence intervals (CIs) of the mediation proportions were calculated using the delta method (40).

#### MR sensitivity analyses

In UVMR, three sensitivity analyses including the weighted median (41), MR-Egger (42), and Mendelian randomization pleiotropy residual sum and outlier (MR-PRESSO) (43) analyses were performed to validate the robustness of the IVW results on the basis of different assumptions. The weighted median method was employed to ensure consistent estimates when more than 50% of the analysis weight is attributed to valid genetic instruments (41). Meanwhile, the weighted mode method provides an unbiased estimate under the condition that the SNPs contributing to the most substantial cluster are valid (44). The MR Egger method serves as a powerful tool for detecting bias resulting from directional pleiotropy. It accomplishes this by scrutinizing the intercept term, where a significant deviation from zero (signified by a *p*-value for Egger intercept < 0.05) points to the existence of directional pleiotropic bias (42). We also conducted an evaluation of directional pleiotropic bias using the MR PRESSO method. This approach identifies outlying SNPs that may exhibit horizontal pleiotropy and assesses whether the exclusion of these outlying SNPs impacts the causal estimates (43). All analyses were carried out using the R software version 4.1.2, along with the TwoSampleMR (45), Mendelian Randomization (41), and MRPRESSO (43) R packages.

## Results

There were strong bidirectional causal associations between education, intelligence, and cognition (Supplementary Table S2). The F-statistic for each genetic variant was above 10, suggesting a good strength of used genetic instruments (Supplementary Table S3).

### Education and gastrointestinal diseases

**Figure 2:**
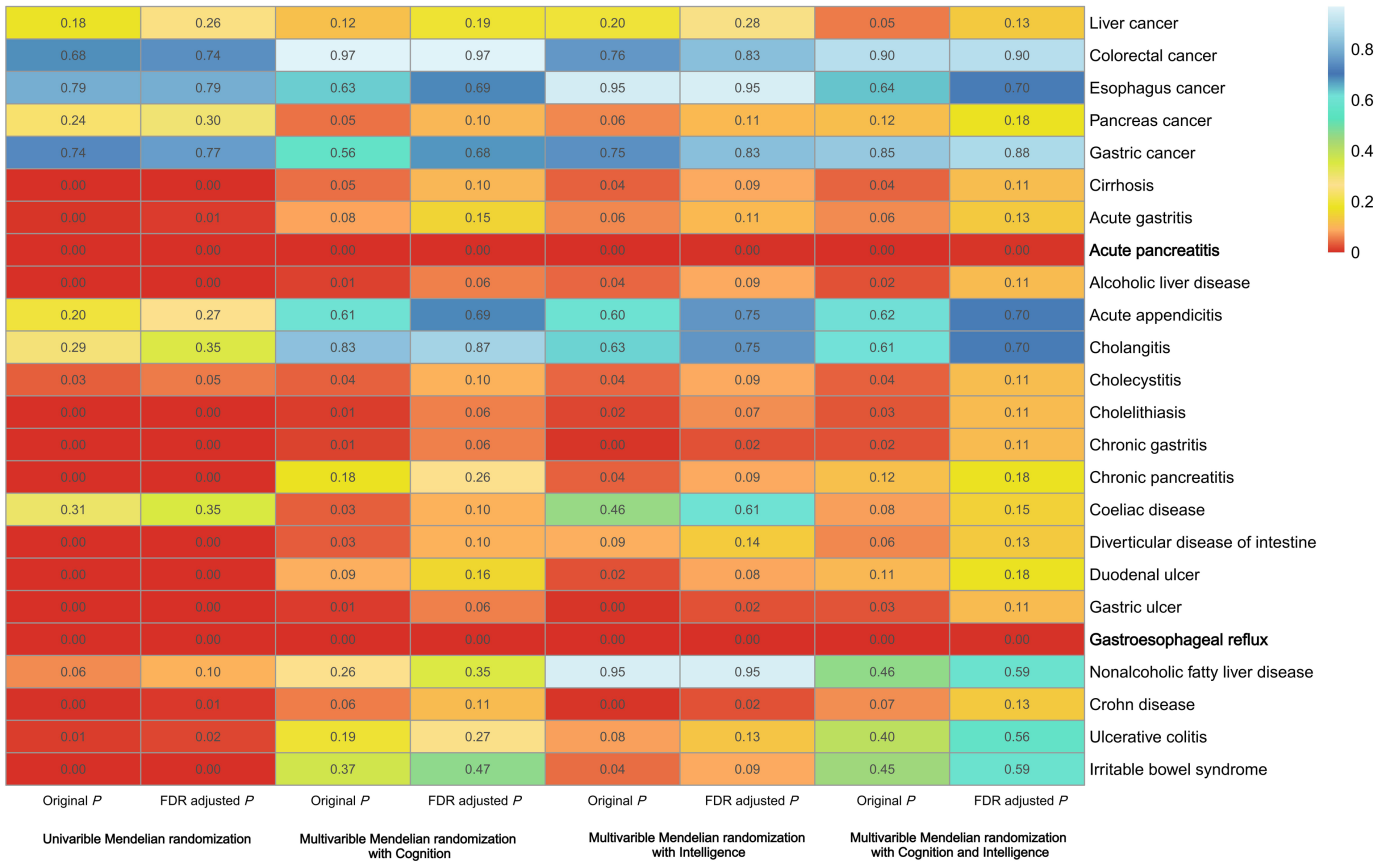
Summary of the relationship between education and 24 gastrointestinal diseases.

In UVMR analyses education was associated with 15 out of the 24 gastrointestinal diseases (Figure 2). Specifically, education was possibly associated with 5 upper gastrointestinal diseases: acute gastritis (OR, 0.662; 95% confidence interval [CI], 0.499–0.879; *p* = 0.004), chronic gastritis (OR, 0.685; 95%CI, 0.586–0.801; *p* = 2.13 × 10^−6^), duodenal ulcer (OR, 0.545; 95%CI, 0.426–0.698; *p* = 1.40 × 10^−6^), gastric ulcer (OR, 0.616; 95%CI, 0.511–0.743; *p* = 3.93 × 10^−7^), gastroesophageal reflux (OR, 0.644; 95%CI, 0.580–0.714; *p* = 8.94 × 10^−17^); 4 lower gastrointestinal diseases: diverticular disease of intestine (OR, 0.766; 95%CI, 0.688–0.853; *p* = 1.22 × 10^−6^), Crohn disease (OR, 0.770; 95%CI, 0.640–0.920; *p* = 0.004), ulcerative colitis (OR, 0.820; 95%CI, 0.710–0.950; *p* = 0.01), Irritable bowel syndrome (OR, 0.660; 95%CI, 0.560–0.770; *p* = 1.99 × 10^−7^); 6 hepatobiliary and pancreatic diseases: cirrhosis (OR, 0.592; 95%CI, 0.468–0.749; *p* = 1.25 × 10^−5^), acute pancreatitis (OR, 0.529; 95%CI, 0.438–0.637; *p* = 2.41 × 10^−11^), alcoholic liver disease (OR, 0.488; 95%CI, 0.365–0.652; *p* = 1.23 × 10^−6^), cholecystitis (OR, 0.786; 95%CI, 0.630–0.981; *p* = 0.033), cholelithiasis (OR, 0.753; 95%CI, 0.681–0.834; *p* = 4.17 × 10^−8^), chronic pancreatitis (OR, 0.522; 95%CI, 0.409–0.666; *p* = 1.71 × 10^−7^). An indication of horizontal pleiotropy was observed in the analysis of acute gastritis (*P* for MREgger intercept < 0.05; Supplementary Table S4). When performing MR-PRESSO, some outliers were detected in Cholelithiasis, Diverticular disease of the intestine. However, after removing these outliers, the associations remained robust and statistically significant (Supplementary Table S4). Following FDR correction, education exhibited significant associations with 14 out of the 24 gastrointestinal diseases, although the relationship between cholecystitis and educational attainment was no longer statistically significant (Supplementary Table S4).

In the multivariable MR analysis, with adjustments for intelligence, cognition, and both of them respectively, the association between education and 8 gastrointestinal diseases, remained statistically significant (cirrhosis, acute pancreatitis, alcoholic liver disease, cholecystitis, cholelithiasis, chronic gastritis, gastric ulcer, and gastroesophageal reflux). However, following FDR correction, only 2 gastrointestinal diseases (acute pancreatitis and gastroesophageal reflux) retained a statistically significant association with education (Supplementary Table S4).

### Intelligence and gastrointestinal diseases

In UVMR analyses, intelligence was found to be negatively associated with 9 out of 24 gastrointestinal diseases. These included 2 upper gastrointestinal diseases (duodenal ulcer and gastroesophageal reflux), 2 lower gastrointestinal diseases (diverticular disease of the intestine and irritable bowel syndrome), and 5 hepatobiliary and pancreatic diseases (pancreatic cancer, cirrhosis, acute pancreatitis, alcoholic liver disease, and cholelithiasis; Supplementary Table S5). After FDR correction, duodenal ulcer was no longer causally associated with intelligence, leaving only 8 gastrointestinal diseases associated with intelligence.

Then we performed three MVMR analyses after adjusting for education, cognition and both of them, respectively. However, none of the gastrointestinal diseases showed a statistically significant association with intelligence in any of the analyses, suggesting that intelligence may not be a dominant factor in gastrointestinal diseases (Supplementary Table S6).

### Cognition and gastrointestinal diseases

Similarly, we first performed UVMR analyses and found that cognition was negatively associated with 10 of the 24 gastrointestinal diseases. Among these, 3 were upper gastrointestinal diseases: chronic gastritis, duodenal ulcer, and gastroesophageal reflux; 2 were lower gastrointestinal diseases: diverticular disease of intestine and irritable bowel syndrome; and 5 were hepatobiliary and pancreatic diseases: pancreas cancer, acute pancreatitis, alcoholic liver disease, cholelithiasis, and chronic pancreatitis (Supplementary Table S7).

After FDR correction, only 6 gastrointestinal diseases remained to be associated with cognition. Acute pancreatitis, alcoholic liver disease, diverticular disease of the intestine, and duodenal ulcer were excluded (Supplementary Table S8).

Similar to intelligence, in three MVMR analyses adjusted for education, intelligence and both of them respectively, no gastrointestinal disease showed a statistically significant association with cognition in each analysis. Therefore, it could be concluded that cognition does not play a significant role in influencing gastrointestinal diseases either.

### Mediation MR analysis

Given that education emerged as a primary factor in gastrointestinal diseases among education, intelligence, and cognition, we conducted a two-step MR analysis to explore the mediating pathway from education to 2 gastrointestinal diseases (acute pancreatitis and gastroesophageal reflux) that were significantly associated with education and 6 gastrointestinal diseases (Cirrhosis, Alcoholic liver disease, Cholecystitis, Cholelithiasis, Chronic gastritis, Gastric ulcer) that had a suggestive association with education. Out of the 14 candidate mediators, 9 risk factors met the screening criteria and were included in the mediation MR analyses (Supplementary Table S9).

#### Effect of education on mediators

In UVMR, each 1-SD higher genetically determined education level was associated with lower smoke initiation (IVW β, −0.28 SD; 95% CI, −0.34 to −0.23), fewer cigarettes per day (−0.31; −0.34 to −0.24), lower smoke index (−0.17; −0.19 to −0.15), lower fat intake (−0.06; −0.09 to −0.03), lower body fat percentage (−0.18; −0.21 to −0.15), lower BMI (−0.21; −0.25 to −0.17), lower hip circumference (−0.13; −0.17 to −0.09), lower waist circumference (−0.17; −0.27 to −0.01), lower waist-to-hip ratio (−0.19; −0.26 to −0.12), lower risk of depression (IVW OR, 0.81 SD; 95%CI, 0.77–0.86). Genetic instrumental variables of education showed persistent heterogeneity with those of mediators and pleiotropy with smoke index (Supplementary Table S10). Although MR-PRESSO several outliers, the relationship between education and each mediator remained significant after removing outliers. We also conducted a Steiger test between education and each mediator, and the results showed that there was no reverse causality for each mediator on education (Supplementary Table S11).

#### Effect of mediators on gastrointestinal diseases

In order to screen the mediators for each of 8 gastrointestinal diseases, we explored the possible association between 9 mediators and 8 gastrointestinal diseases by UVMR analysis (Supplementary Table S12). In MVMR analysis, after adjusting for education, we observed that depression and each 1-SD increase in genetically determined lifestyle factors, including BMI and body fat percentage, were causally associated with a 1.13–2.09 increased risk of gastroesophageal reflux. Additionally, a higher risk for gastric ulcer was observed in relation to depression, increased smoke index, smoking initiation, and BMI, with odds ratios ranging between 1.11 and 2.05.

Several factors, including daily cigarette consumption, hip circumference, and specific body metrics, were linked to cholelithiasis (OR, 1.14–1.83) and cholecystitis (OR, 1.31–2.30). Furthermore, there were similar causal associations of mediators (drinks per week and smoking Initiation) with higher odds of alcoholic liver disease, mediators (drinks per week, depression, smoking initiation, BMI, body fat percentage) with a higher risk of acute pancreatitis and mediators (drinks per week, smoking initiation, BMI) with a higher risk of cirrhosis (Supplementary Table S13).

#### Mediation effect of each mediator

**Figure 3:**
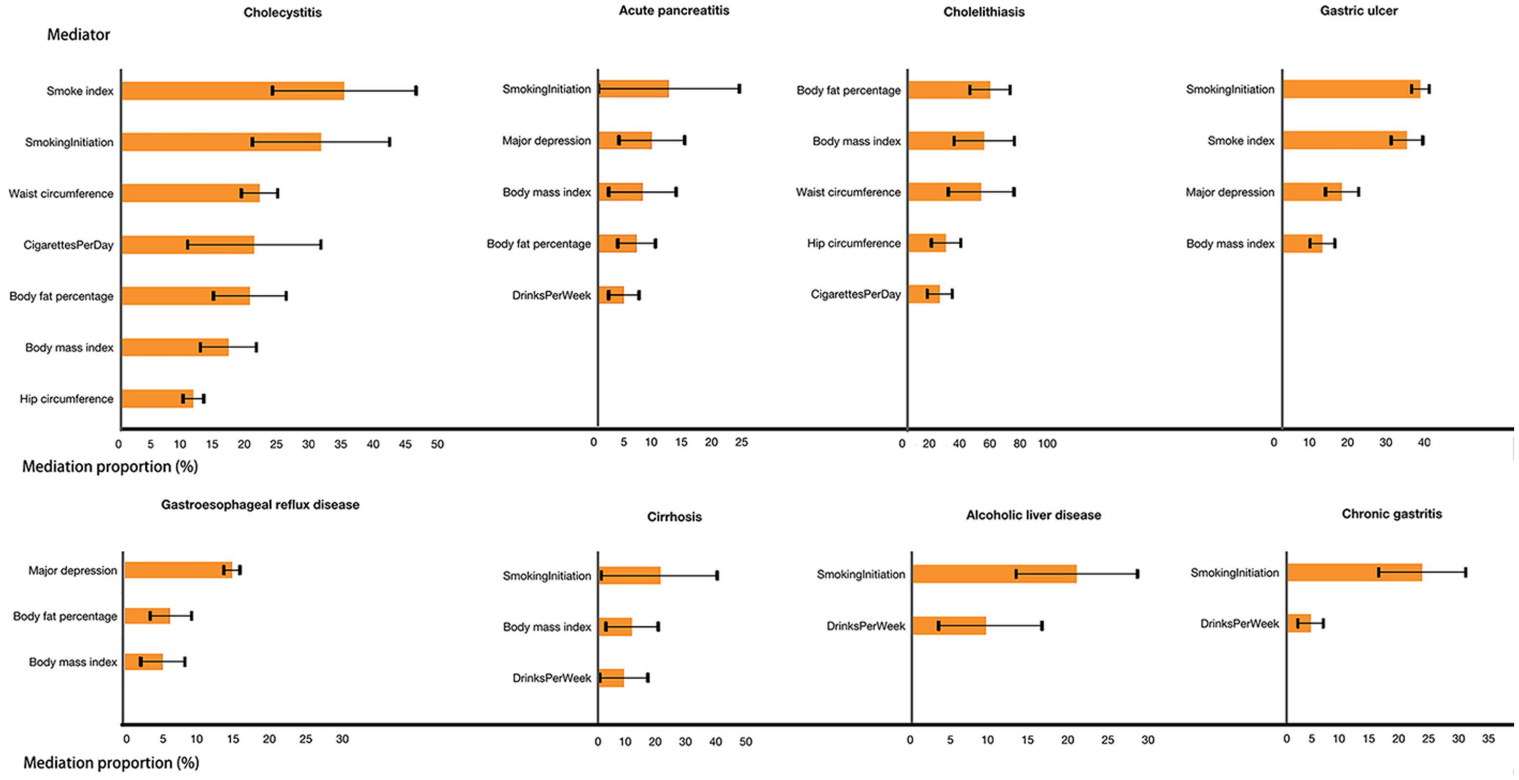
Effect of each mediator in the association between education and gastrointestinal diseases.

Concerning gastroesophageal reflux, major depression, BMI, and body fat percentage mediated 14.6, 5.2, and 6.3% of the overall effect between education and the disease, respectively. For gastric ulcer, major depression, smoke index, smoking initiation, and BMI, as significant mediating factors, contributed 15.6, 32.4, 35.9, and 10.5%, respectively, to the overall effect. Chronic gastritis demonstrated mediation through drinks per week (4.0%) and smoking initiation (22.5%). Furthermore, cholelithiasis exhibited mediation via cigarettes per day (20.7%), hip circumference (24.9%), BMI (49.9%), body fat percentage (53.7%), and waist circumference (47.9%). For cholecystitis, mediating factors included cigarettes per day (20.6%), smoke index (34.5%), smoking initiation (30.9%), hip circumference (12.0%), BMI (16.6%), body fat percentage (19.9%), and waist circumference (21.4%). Alcoholic liver disease revealed mediation through drinks per week (9.1%) and smoking initiation (20.3%). Acute pancreatitis displayed mediation via drinks per week (4.2%), major depression (8.8%), smoking initiation (11.6%), BMI (7.3%), and body fat percentage (6.3%). Finally, cirrhosis demonstrated mediation via drinks per week (8.3%), smoking initiation (20.4%), and BMI (11.0%; Figure 3).

## Discussion

In this study, we investigated the relationship between education and 24 gastrointestinal diseases, as well as their potential mediators using MR analysis. Independent of the effects of intelligence and cognition, increased years of education reduced the risk of 8 gastrointestinal diseases. Furthermore, we selected 14 potential mediators associated with the development of gastrointestinal diseases, of which 9 were selected as qualified mediators. The proportion of mediator effects exerted by their locations in different gastrointestinal diseases was calculated.

The current MR study confirmed the findings of previous epidemiologic studies that elevated education reduces the risk of developing reflux esophagitis (46, 47), gastric ulcers (48, 49), alcoholic liver (50), and cirrhosis (6). Meanwhile, our MR study was consistent with previous MR studies, and our MR also found that education was associated with a reduced risk of acute pancreatitis (51) and reflux esophagitis (52). While most observational studies have found that education reduces the risk of many gastrointestinal cancers, our MR study found no such association. Rota M et al. included 9,773 cases of gastric cancer and 24,373 controls from 25 studies in Europe, Asia, and the Americas (53) and found that the incidence of gastric cancer was lower in highly educated populations compared with those of low education levels OR 0.60 (95% CI, 0.44–0.84). The inconsistency in the conclusions between the two studies may be attributed to the relatively small sample size of gastric cancer patients in our study (1,307 cases). Regarding colon cancer, our findings were consistent with observational studies that educational level was not associated with the risk of colon cancer. A large national survey in Finland included 77,614 patients with colon cancer, and although the incidence of colon cancer was higher in the male population with higher educational levels between 1976 and 2004, there was no statistical difference in colon cancer incidence by educational level after 2005 (54). The validity of this conclusion was further supported by the fact that our study included a Finnish population as well. However, there are not enough observational studies to verify the association between education and liver, pancreatic, and esophageal cancers. Overall, our MR study refined the gastrointestinal classification and provided further validation of the relationship between education and several gastrointestinal diseases. The association between education and chronic gastritis, cholecystitis, and gallstones was a new finding and requires further confirmation.

This study explored the mediating role of education in the association with gastrointestinal diseases. 14 candidate mediators were selected after a rigorous screening, and 9 mediators were selected. Notably, although smoking only accounted for only 2 of the 9, it mediated the causal effect of education on 7 other diseases with mediation proportion > 10%, except for gastroesophageal reflux (Supplementary Table S12). This suggested that smoking played an important mediating role in the relationship between education and gastrointestinal diseases. Several studies from different countries and regions around the world showing that smoking behavior was associated with socioeconomic inequality (55–60), with educational inequality being one of the important factors. Additionally, the increased risk of several gastrointestinal diseases associated with smoking has also been reported in previous studies (61–64). The fact that increased education reduces the risk of gastrointestinal diseases may be due to the reduction of unhealthy lifestyle habits such as smoking and alcohol consumption. Considering the mediating effect of smoking in the relationship between education and gastrointestinal diseases, it can be inferred that alcohol consumption is similar to smoking. 4 out of the 9 identified mediators were adiposity traits, which played a similar role in mediating the association between education and various gastrointestinal diseases. Our findings suggested that preventive strategies targeting these 3 mediators may contribute, in part, to the protective effect of education against multiple gastrointestinal diseases. Our MR study has some implications for the development of educational policies: while pursuing the improvement of education, emphasis should be placed on the development of good living habits, as this can prevent the occurrence of many diseases. A strong body can contribute to the improvement of personal learning, creating a positive feedback loop.

Although a number of studies have reported lower rates of depression in populations with higher levels of education (65–67), the current prevalence of depression in the overall population continues to increase (68), and physical health problems caused by depression still deserve our attention. The effects of depression on gastrointestinal diseases can be elucidated through several physiological mechanisms. It begins with the activation of the Hypothalamic–Pituitary–Adrenal axis in response to chronic stress, resulting in elevated levels of pro-adrenocorticotropic hormones and cortisol (69). Excessive cortisol can induce or exacerbate peptic ulcers by interfering with tissue repair, increasing gastric acid and pepsin levels, and reducing gastric mucosal secretion. This ultimately compromises the gastric mucosal barrier, contributing to the development of peptic ulcers and gastroesophageal reflux. Moreover, elevated cortisol can disrupt the balance of the intestinal microbiota and lead to intestinal as well as extraintestinal diseases (70).

Our study pioneered the use of Mendelian Randomization (MR) to identify the causal effects of education on various gastrointestinal diseases, independently considering factors such as intelligence and cognition. In addition, we aimed to identify causal mediators in the pathways associated with education and gastrointestinal disorders. Our study has several advantages, first the exposure, outcome and mediator sources are different with minimal overlap in GWAS datasets among the three to ensure the lowest type 1 error rate. Second, we set strict criteria for mediator screening to minimize reverse causality of mediators on education and to ensure the credibility and plausibility of the model we constructed to account for mediator effects. Thirdly, our study included 24 common gastrointestinal diseases as outcomes. This design allowed us to explore the connection between education and various gastrointestinal diseases more thoroughly, providing a comprehensive perspective. Using multiple diseases as independent outcomes provided more information about associations, and formed a detailed foundation for future research and interventions. This study has some limitations: firstly, our outcome data source is relatively homogenous and we did not include data from UK-biobank due to database permissions. Secondly, although the concordance of the results from multiple sensitivity analyses, horizontal pleiotropy, outliers, and sample overlap—factors that might violate the basic MR assumptions—did not substantially influence our causal estimates, the causal associations should be interpreted with caution. This is because several assumptions of the methods are untestable, and the heterogeneity of and residual horizontal pleiotropy might still bias some results. Thirdly, although we included many kinds of risk factors associated with gastrointestinal diseases as mediators, the mediating pathways included in this study are still very limited. Finally, our findings were based on a GWAS primarily conducted among individuals of European ancestry in high-income countries. Therefore, generalizing our findings to other ethnic groups or to low- and middle-income countries requires further investigation.

In conclusion, our study detailed the protective effects of education as a factor independent of intelligence and cognition on multiple gastrointestinal diseases. It also elucidated the mediating role of common gastrointestinal risk factors, such as obesity, smoking, alcohol consumption, and depression, between education and multiple gastrointestinal diseases.

## Data availability statement

The original contributions presented in the study are included in the article/Supplementary material, further inquiries can be directed to the corresponding author.

## Ethics statement

Ethical approval was not required for the study involving humans in accordance with the local legislation and institutional requirements. Written informed consent to participate in this study was not required from the participants or the participants’ legal guardians/next of kin in accordance with the national legislation and the institutional requirements.

## Author contributions

MG: Conceptualization, Methodology, Writing – original draft, Writing – review & editing. MW: Data curation, Visualization, Writing – review & editing. DW: Investigation, Writing – review & editing. TX: Data curation, Methodology, Writing – review & editing. XW: Supervision, Writing – original draft, Writing – review & editing.
